# Cloning and Expression of Class I Chitinase Genes from Four Mangrove Species under Heavy Metal Stress

**DOI:** 10.3390/plants12152772

**Published:** 2023-07-26

**Authors:** Yue-Yue Zhou, You-Shao Wang, Cui-Ci Sun, Jiao Fei

**Affiliations:** 1 State Key Laboratory of Tropical Oceanography, South China Sea Institute of Oceanology, Chinese Academy of Sciences, Guangzhou 510301, China; m17889984171@163.com (Y.-Y.Z.); scuici@scsio.ac.cn (C.-C.S.); 2 Daya Bay Marine Biology Research Station, Chinese Academy of Sciences, Shenzhen 518121, China; 3 Innovation Academy of South China Sea Ecology and Environmental Engineering, Chinese Academy of Sciences, Guangzhou 510301, China; 4 University of Chinese Academy of Sciences, Beijing 100049, China

**Keywords:** mangrove plants, chitinase, cloning, gene expression

## Abstract

Chitinases are believed to act as defense proteins when plants are exposed to heavy metal stress. Typical Class I chitinase genes were cloned from *Bruguiera gymnorrhiza*, *Rhizophora stylosa*, *Kandelia obovata*, and *Avicennia marina* using the methods of reverse-transcription–polymerase chain reaction and rapid amplification of cDNA ends. All four cDNA sequences of chitinase from the mangrove plants were 1092 bp in length and consisted of an open reading frame of 831 bp, encoding 276 amino acids. However, there were differences in the sequences among the four mangrove species. Four gene proteins have a signal peptide, are located in the vacuole, and belong to the GH19 chitinase family. The sequence of chitinase was highly similar to the protein sequences of *Camellia fraternal* chitinases. A real-time polymerase chain reaction was used to analyze the chitinase expressions of the above four mangrove species exposed to different concentrations of heavy metal at different times. The gene expression of chitinase was higher in *Bruguiera gymnorrhiza* leaves than in other mangrove plant species. With an increase in heavy metal stress, the expression level of *Bruguiera gymnorrhiza* increased continuously. These results suggest that chitinase plays an important role in improving the heavy metal tolerance of mangrove plants.

## 1. Introduction

The mangrove wetland is an important ecosystem in the intertidal zone of tropical and subtropical coasts and possesses four notable characteristics: high productivity, a high return rate, high decomposition rate, and high temperature resistance, making it one of the most unique marine ecosystems in the world [1,2]. It has important environmental functions and ecological benefits in terms of wind and wave protection, water purification, biodiversity protection, food supply, and habitat [1,3]. With the rapid development of modern industry, heavy metal pollution in offshore environments around the world is becoming more and more serious due to its toxicity and decades-long persistence in the water environment [4,5]. Mangrove plants also have a certain tolerance for heavy metals when they live in seriously polluted environments for a long time. The adaptation mechanisms of mangrove plants to heavy metals include the absorption and efflux of heavy metals [6], regionalization [7], chelation of organic compounds [8], scavenging of free radicals caused by heavy metal stress through various antioxidant defense systems [9,10,11,12], and induced expressions of some defense genes [13,14,15,16].

It has been confirmed that chitinase is a pathogenesis-related protein (PR protein) in plants and has been divided into at least five classes (I, II, III, IV, and V) based on sequence similarities [17,18]. Plant chitinases have a wide range of physiological activities and play an important role in plants. Some studies show that type I endocytic chitinase, which hydrolyzes linear polysaccharide chains of chitin and peptidoglycan, plays important roles in the defense against pathogenic bacteria and fungi [19]. Some studies show that the gene expression of plant chitinases is tissue-specific and involved in the developmental regulation of plants. For example, chitinases are involved in the developmental regulation of muskmelon [20]. Most chitinases are also induced by some biological or non-biological factors, such as mechanical damage, chitin, ethylene, salicylic acid, heavy metals, UV, osmotic pressure, low temperature, and drought stress [21]. In normal conditions, chitinase gene expression is very low or not highly expressed in most plants. When plants are also infected by pathogenic fungi, bacteria, or viruses or subjected to mechanical trauma or ethylene treatment, chitinase expression activities are greatly increased. One review focused on the current state of knowledge on the role of chitinase in plants’ tolerance to heavy metals. The chitinase may alter the kinetics and permeability of the cell wall and affect the metal binding and immobilization capacity of the cell wall [22]. In addition, it can generate signaling molecules that trigger further defense responses [23]. Although the exact role of these enzymes in metal defense is not known, they appear to be stable components of plant defense against metal stress [24,25]. Transgenic plants overexpressing these genes have been shown to exhibit increased tolerance to heavy metals [26]. At the same time, such plants are often induced to express glucanase (EC3.2.1.39), which plays a key role in plant disease resistance and defense responses. It was found that there were three types of chitinase in peas treated with 3 mg/kg of Cd sand for one week, and the results showed that the chitinase gene expression of those plants was higher than that of the control [27]. Mycorrhizal and non-mycorrhizal peas were cultured in 100 mg/kg of Cd sand for 3 weeks, and gene expression analysis showed that the expression amount of chitinase, heat shock protein, metallothionein, and glutathione synthetase was significantly higher than that in the control group without Cd treatment [28]. It has been shown that chitinase is involved in lignin accumulation and that lignin is important to heavy metal fixation accumulation [29]. It has also been shown that chitinase is associated with the development of the plant root cell wall, which is an important site of heavy metal treatment. Chitinases are involved in processes related to heavy metal resistance in plants [30]. Chitinase genes in faba bean (*Viciafaba*), barley, maize, and soybean are triggered by lead, arsenic, and cadmium, suggesting that this enzyme plays a role in preventing heavy metal toxicity [25,31]. All plants will confront biological and abiotic stress during their growth, and heavy metal pollution is a form of abiotic stress. It has been reported that heavy metal ions can induce oxidative stress in plants, and it has been suggested that the accumulation of reactive oxygen species in plants under heavy metal stress leads to the accumulation of H_2_O_2_. The accumulation of H_2_O_2_ diffused into the plant and induced the transcription of the chitinase gene, the accumulation of the corresponding mRNA, and a corresponding increase in enzyme activity [28,32,33]. Many chitinase genes from terrestrial plants such as tobacco [34], potato [35], pear [36], rice [37,38], etc., have been cloned. These genes can be triggered by various stressors. Since chitinase, as a defense protein, may not directly participate in metal binding, why does it also play an important role in the metal tolerance of mangrove plants? The answer to this question is not clearly known at present. Class Ⅰ and III chitinase genes were first cloned from *A. corniculatum* and *A. marina* in our earlier research [39,40] and will be needed for further research on mechanisms of chitinase genes in mangrove plants [2].

Four mangrove species were selected for the experiment: *Bruguiera gymnorrhiza*, *Rhizophora stylosa*, *Kandelia obovata*, and *Avicennia marina*. Why did the researchers choose these plant species? Based on previous experimental studies, it was found that mangrove plants are tolerant to heavy metal stressors. We obtained physiological parameters from seedlings following heavy metal stressors, so four common mangrove plants were chosen [12]. These were subjected to chitinase gene isolation because we wished to understand the functional role of the chitinase gene under heavy metal stress by first isolating the chitinase gene and understanding the basic sequence and protein space structure. Generally, the structure determines the functional role. In order to know more about the molecular mechanisms of heavy metal tolerance in mangrove plants, we cloned and sequenced the cDNA that encodes the CHI from mangrove plant seedlings’ young leaves. Some studies have shown that other mangrove species (for example, *Aegiceras corniculatum*) [39] are already known (since 2015) to induce the expression of a Class I chitinase via cadmium stress. However, the gene sequences are distinct for different species, and the patterns of expression are yet to be explored.

In the paper, four chitinase genes of Class I chitinase were first cloned using RT-PCR(reverse-transcription–polymerase chain reaction) and RACE (rapid amplification of cDNA ends) methods from *Bruguiera gymnorrhiza*, *Rhizophora stylosa*, *Kandelia obovata*, and *Avicennia marina*. We also elucidated the mRNA expression pattern of CHI I in response to heavy metal stress using qPCR. In view of their important role in plant disease resistance and stress resistance, further studies on the mechanisms of heavy metals will have theoretical significance and potential practical value.

## 2. Results

### 2.1. The Full-Length cDNA of CHI I Gene Cloning

**Figure 1:**
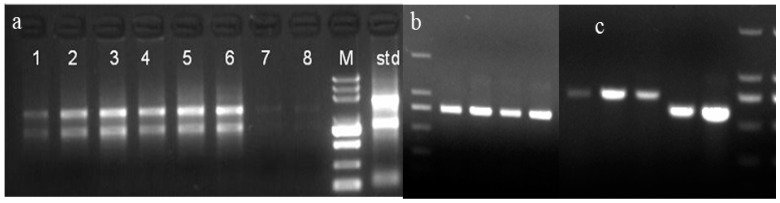
Agarose gel electrophoresis of total RNA (**a**), CHI I fragment (**b**), and PCR products of 3′ or 5′ RACE (**c**); 1–8: electrophoretic bands of samples of RNA; M: DNA Marker DL5000; std: electrophoretic bands of standard samples of RNA.

Evident 28S and 18S bands were seen, as illustrated in Figure 1a, suggesting excellent RNA integrity. The OD 260/OD 280 ratios of the total RNA samples were between 1.8 and 2.2, according to the UV detection data, suggesting that the RNA purity was high. As a result, the suggested RNA is of high purity and good quality, meeting the standards set by the following experiments. With the cDNA of the leaf as the template, an intermediate fragment of about 750 bp was obtained via amplification with degenerate primers (Figure 1b). Blast was performed after sequencing, and the results showed that the fragment was highly homologous to the chitinase gene of other plants (84.73–74.72%), indicating that the fragment was the intermediate fragment of the CHI I gene. According to the amplified intermediate fragment sequence, two pairs of primers for the rapid amplification of the 3′ and 5′ ends were designed. After the first and second cycles of 3′ and 5′ RACE PCR, the 3′ and 5′ end-specific fragments of the gene were amplified (Figure 1c). The sequencing results of these fragments were spliced using MEGA-X software and submitted to NCBI for Blast homology analysis. Finally, we confirmed a correctly encoded nucleotide sequence.

### 2.2. Sequence and Structure Analysis of the Full-Length cDNA Sequence of CHI I

Cloning and characterization analysis of the full-length cDNA sequence indicated that the full-length cDNA fragment encodes a chitinase gene, designated as *BgChi*, *KoChi*, *AmChi*, and *RsChi* (Figure S1). All four cDNAs were 1092 bp, including an 831 bp open reading frame encoding a protein of 276 amino acids. There are different sequences among the four species, with 6–30 different bases (Figure S2). In Figure S2, red markers show the differences in four full-length genes. *BgChi* has a predicted molecular mass of 29.50 kDa and a pI of 4.47. *KoChi* has a predicted molecular mass of 29.59 kDa and a pI of 4.74. *AmChi* has a predicted molecular mass of 25.57 kDa and a pI of 4.66. *RsChi* has a predicted molecular mass of 29.47 kDa and a pI of 4.65. Stable proteins are found when the stability coefficient is less than 40; unstable proteins are found when the stability coefficient is greater than 40; hydrophilic proteins are found when the hydrophilic coefficient is negative; and hydrophobic proteins are found when the hydrophilic coefficient is positive (Table 1). The data show that the Chi I polypeptide is a stable hydrophilic protein. The amino acid components of the four species are in the top three, which are glycine, serine, and alanine. Although there are differences in the physical and chemical data, the difference is small.

**Figure 2:**
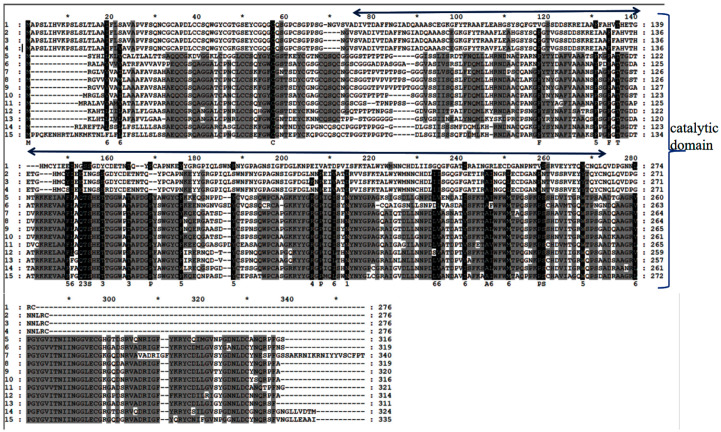
Multiple-sequence alignment analysis of amino acid sequences between type I chitinase gene and other plants.

**Figure 3:**
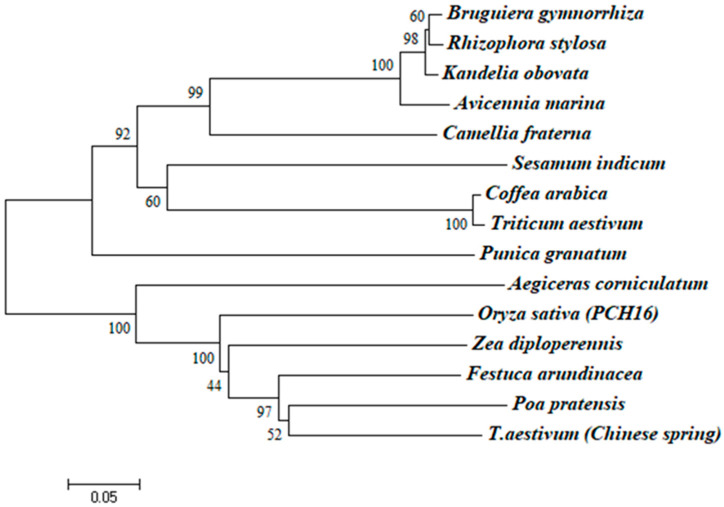
Phylogenetic tree of the CHI I. Multiple alignments of the sequences of CHI I and other selected plant chitinases were performed using MEGA 6.

**Figure 4:**
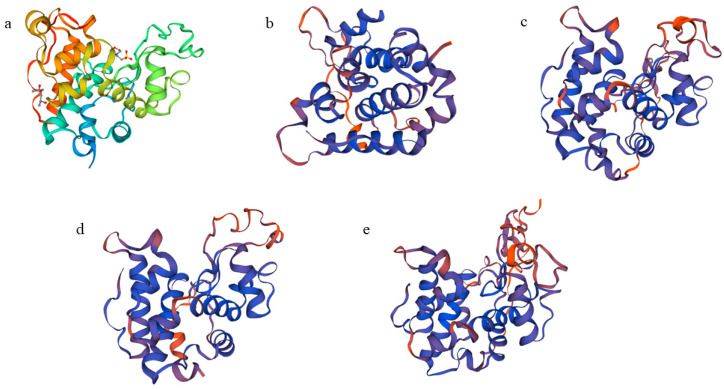
The molecular model of CHI I ((**a**) *Oryza sativa*. (**b**) *Bruguiera gymnorrhiza*. (**c**) *Kandelia obovata*. (**d**) *Avicennia marina*. (**e**) *Rhizophora stylosa*).

**Figure 5:**
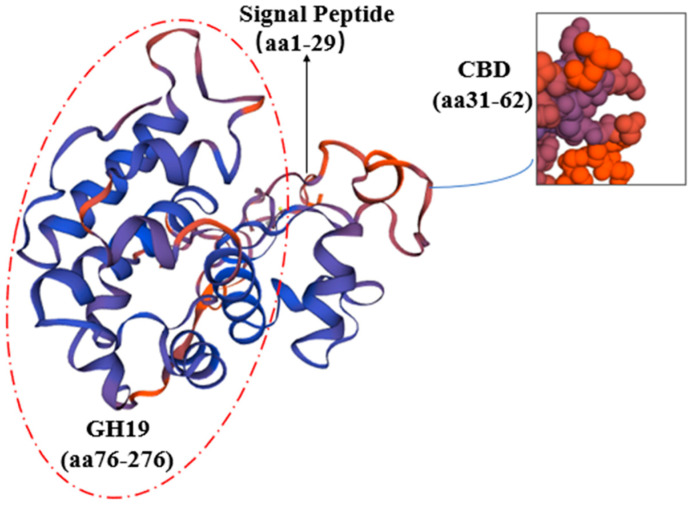
Specific division of chitinase’s spatial structure.

A comparison of the CHI I amino acid sequence of proteins from mangrove plants revealed that CHI I shared a high degree of similarity to the Class I chitinases of other plants (85.11–76.95% similarity) in the GenBank database. The CHI Ⅰ gene was cloned from 4 mangrove plants, and the CHI genes from other 11 plants were compared for homology. The 11 plants were *Aegiceras corniculatum* (AFK26307.1), *Oryza sativa* (Z29961.1), *Poa pratensis* (AF000964.1), *T. aestivum* (Chinese spring) (X76041.1), *Triticum aestivum* (AY437443.1), *Festuca arundinacea* (EU837265.1), *Zea diploperennis* (AY532761.1), *Camellia fraterna* (MG720756.1), *Coffea arabica* (XM_027261888.1), *Sesamum indicum* (XM_011093269.2), and *Punica granatum* (XM_031550186.1). The 4 chitinase proteins and 11 other plant chitinases are more conserved in the CBD (chitin-binding region), which is mainly responsible for binding to chitin and contributing to the better hydrolysis of the catalytic domain (Figure 2). The 4 mangrove plants’ CHI Ⅰ proteins, along with those of 11 other plants, were used to construct the phylogenetic tree via the MEGA6 software (Figure 3). BgChi showed very close homology to KoChi and RsChi in Figure 3. Mangrove plants are most closely related to *C. fraterna*, followed by S. indicum, and relatively distantly related to *P. pratensis* and *T. aestivum*. The amino acid sequence analysis-based kinship determination was the same as that based on traditional evolutionary kinship determination. Furthermore, the protein was predicted to be located in vacuoles according to Plant-mpLoc [41]. Based on SWISS-MODEL [42] analysis, ribbon cartoons and space-filling models of CHI I are presented in Figure 4. The GH 19 chitinase from rice (*Oryza sativa*; SMTL id: 3iwr.i.A) [43] was determined as a modeling template (Figure 4). All four genes have the same sequence number of 1–831 nucleotides that can be translated into proteins. Four 3D models of Chi all contained a seven-α-helix structure and some random coil structures (Figure 4). The predicted proteins all had a structure typical of Class I chitinases, consisting of a signal peptide region at its N-terminus (amino acids 1–29), a chitin-binding domain (CBD) (amino acids 31–62), and a glycosyl hydrolase catalytic domain (GH19) (amino acids 76–276) (Figure 4 and Figure 5).

### 2.3. CHI I mRNA Expression in Leaf in Response to Heavy Metal

**Figure 6:**
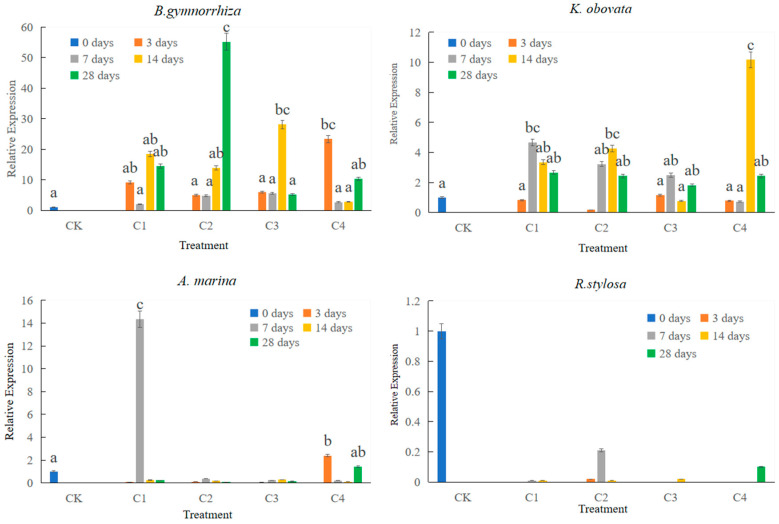
Expression of *CHI* gene in leaves of four species in response to heavy metal stresses using real-time quantitative PCR analysis. Data are the means ± standard of three separate individuals. Different lowercase letters on bars indicates significant difference (*p* < 0.05).

These heavy metals were Cu, Pb, and Cd in the experiment. To realize the expression patterns of *CHI I* induced by heavy metal stress, total RNA was isolated from four mangrove seedling leaves after heavy metal stimulation. The effects of heavy metal on the expression of CHI I mRNA in leaves are presented in Figure 6. The real-time quantitative PCR (qPCR) results revealed that the expression patterns of four mangrove species were very different. Chitinases were expressed in *B. gymnorrhiza*, *K. obovata* and *A. marina* under heavy metal stress. Under heavy metal stress, the gene expression of *CHI I* was highly induced in the *B. gymnorrhiza* leaves, while the expression level of *R.stylosa* was basically zero. The highest gene expression of *B. gymnorrhiza* was 55.23 times that of the control group. The highest gene expression level of *K. obovata* was 10.17 times that of the control group. The highest gene expression of *A. marina* was 14.36 times that of the control group. With the increase in heavy metal concentration, the gene expression of *B. gymnorrhiza* increased first and then decreased. The gene expression of *K. obovata* increased with the increase in heavy metal concentration (Figure 6).

After 3 days of heavy metal stress, *CHI I* gene expression was first induced in *B. gymnorrhiza*. After 7 days of heavy metal stress, the expression of *A. marina* was the highest. After 28 days of heavy metal stress, the expression of *B. gymnorrhiza* was the highest. With the increase in heavy metal stress time, the expression level of *B. gymnorrhiza* increased continuously, and the gene expression level of *K. obovata* remained stable (Figure 6).

## 3. Discussion

### 3.1. Cloning and Structural Characterization Analysis of CHI I

Plant chitinase precursors generally contain an N-terminal signal region, a catalytic region, and a C-terminal extension region. Some are chitin-binding domain (CBD)-rich in cysteine after the N-terminal signal region, which is connected with the catalytic region by the variable cross-linking region [44]. The GH19 family consists of all I, II, and IV chitinases [45]. In this study, chitinase genes (*CHI I*) were cloned from *B. gymnorrhiza*, *K. obovata*, *A. marina*, and *R. stylosa* for the first time (Figure 2). This was carried out to predict chitinase protein structures, including the signal region, CBD, and GH19 chitinase family catalytic domains using SMART software, and most of them are small molecular proteins with molecular weights ranging from 25 to 35 kDa [46]. In this study, it was found that all four cDNAs were 1092 bp, including an 831 bp open reading frame encoding a protein of 276 amino acids with a molecular weight between 25.57 and 29.59 kDa (Table 1). The results revealed that *BgChi*, *KoChi*, *AmChi*, and *RsChi* were typical Class I chitinases with the characteristic catalytic structure of chitinases via bioinformatic analysis.

The sequences among the four species are different (Figures S1 and S2, Table 1). Compared to *R. stylosa*, there is one amino acid difference in *B. gymnorrhiza*, five amino acid differences in *K. obovata*, and ten amino acid differences in *A. marina* (Figure S2). *BgChi* showed very close homology to *KoChi* and *RsChi* as seen in Figure 3. These results indicated that *B. gymnorrhiza*, *K. obovata*, and *R. stylosa* belong to the same family of *Rhizophora*, while *A.marina* belongs to *Verbenaceae*. The phylogenetic tree analysis indicated that *CHI* had the closest relationship with chitinase in *Camellia fraternal* (75.05% similarity) (Figure 3). The phylogenetic clustering results were more consistent with the traditional morphological classification results. *CHI I* of *A. corniculatum* exhibited very close homology to the Class I chitinase from *Camellia sinensis* (69% similarity) [39]. The results of the multiple-sequence alignments of the amino acid sequences of other plants’ chitinase gene type I are shown in Figure 2.

Iseli et al. studied Class I chitinase genes in tobacco suggesting that CBD was not catalytic and antifungal activity is necessary, but binding chitin was necessary and had an enhanced antibacterial effect [47]. The CBD of Class I chitinases that acted as allergens in avocados and chestnuts may be associated with allergic reactions [48].

Chitinases in plants are encoded by single genes, both secreted outside and localized inside. In this study, the CHI protein was predicted to locate on vacuoles in cells according to Plant-mpLoc [41]. The CHI protein for mangroves is a hydrophilic protein with a signal peptide, and it may be about the possibility of a transmembrane [49]. The C-terminal extension of Class I chitinases has been found to be a vesicular target signal, i.e., it is sufficient to direct mature proteins into the vesicle. The signal peptide controls the protein secretion pathway, locates the protein at a specific location, and is cleaved when the protein is translocated across the membrane [50]. It has been shown that the C-terminal extension is deleted when tobacco Class I chitinase is translocated and the chitinase is secreted into the extracellular space or the culture medium [51].

### 3.2. Expression of CHI I in Leaves in Response to Heavy Metal

Plant chitinases are induced by a series of abiotic stresses, including osmotic stress, salt stress, low-temperature stress, mechanical damage, and heavy metal stress [52]. The Class I chitinase gene was induced via mechanical damage in *Ficuscarica* [53]. Studies have shown that *L. gmelinii* chitinase gene type IV was expressed in root, stem, and leaf tissues, with the highest expression in the stem and the lowest expression in the root [54]. It has been shown that the specific expression levels of chitinase in *Z. bungeanum Maxim* were found to be in the follwing order: stem > fruit > leaf, and the difference in expression levels between stem and leaf reached an extremely significant level (*p* < 0.05) [55]. The chitinase gene sequences of *A. corniculatum* were cloned in our previous research, and the roots and leaves were used as the experimental objects [39,40]. The experimental data showed that the expression level of chitinase gene type I was higher in the leaf than the root after heavy metal treatment, indicating that the expression was different in different tissues [40]. Most people think that choosing roots will be better because roots play a major role in the process of heavy metal resistance, but the expression of genes does not necessarily reflect the same rule, and all the subjects in this experiment are leaves of different mangrove species.

In terrestrial plants, the effects of chitinases have been studied to varying degrees. Stress associated proteins, such as peroxidase and chitinase, were also found to be associated with Hg in the vines [56]. The protein of chitinase may be involved in the decomposition and metabolism of the cell wall macromolecule catabolic process and carbohydrate metabolic process [56]. Plant chitinases not only play a role in metal metabolism but also in the detoxification of excess heavy metals. Heavy metal accumulation can disturb the absorption and distribution of large amounts of elements and trace elements in plants and cause plant death. Due to long-term environmental selection and adaptive evolution, plants have developed tolerance mechanisms to reduce or avoid heavy metal toxicity [57]. Cd treatment could induce the up-regulation of chitinase, heat shock protein (HSP70) and other genes [27]. After three weeks of culture on 100 mg/kg of Cd sand, the gene expression analysis showed that the expression amounts of chitinase, heat shock protein, metallothionein, and glutathione synthase were significantly higher than those in the control group without Cd treatment in Mycorrhizal peas and non-mycorrhizal peas [28]. Chitinase genes in *Vicia faba*, barley, maize, and soy bean were induced by lead, arsenic, and cadmium, indicating that this enzyme could prevent heavy metal toxicity [25,31]. The chitinase was preliminarily cloned from *A. corniculatum* and analyzed via single analysis [40]. In the paper, further research will be conducted on the other four mangrove species treated with a variety of heavy metals to explore the differences among species, and the mechanism of heavy metal resistance in mangrove plants will be further analyzed and discussed.

**Figure 7:**
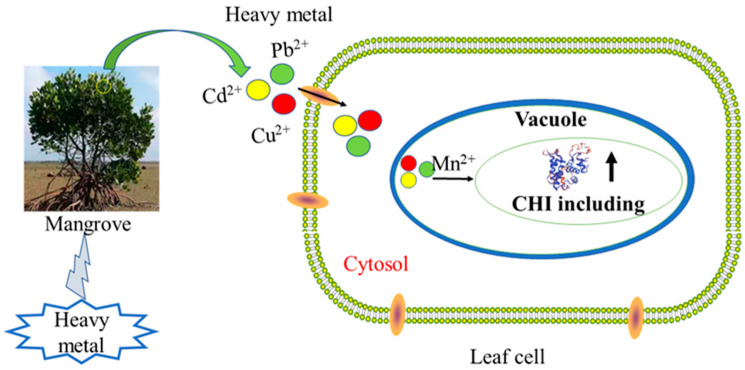
A schematic diagram of the mechanism of chitinase resistance to heavy metals.

Heavy metals (Cu, Cd, and Pb) are important pollutants in the environment, and often exist in nature as compounds of pollution [58]. Under combined pollution, the tolerance mechanism of plants is more complex, and it is more necessary to study the effect of combined pollution on plants and the response of plants to combined pollution [11]. In addition to *R. stylosa*, the *CHI* of the other three mangrove plants was induced by heavy metal stress. The results of this study showed that the expression of *CHI I* was significantly induced in leaves of *B. gymnorrhiza*, *K. obovata*, and *A. marina* under the heavy metals. Real-time quantitative results can be obtained for four mangrove expression patterns that were not the same under heavy metal stress. The maximum expression levels in leaves *B. gymnorrhiza*, *K. obovata*, and *A. marina* were 55.23, 10.17, and 14.36 times that of the control, respectively. The gene expressions of *CHI I* were more highly induced in *B. gymnorrhiza* leaves than in other mangrove species. With the increase in the heavy metal stress time, the expression level of *B. gymnorrhiza* increased continuously. *R. stylosa* was tolerant to heavy metals and had an antioxidant enzyme system [12], while chitinase had little effect. The physiological parameters of different mangrove plant seedlings were being analyzed and described in detail following the application of heavy metal stressors [12]. These experiments have been conducted before and suggest that an antioxidant enzyme system plays an important role in being tolerant to heavy metals. It has been known that chitinases are located in vacuoles in cells, and have a signal peptide region. The signal peptide sequence, which is responsible for guiding proteins into subcellular organelle vacuoles. Plant cell walls and vacuoles are areas rich in heavy metals, which are regionalized and isolated from other organelles to isolate the interference of heavy metals in plant normal metabolism [59]. Chitinase expression was induced when heavy metal entered the leaves, and the expressed proteins may act on metal in vacuoles, which chitinase chelates with heavy metal ions. That may reduce the accumulation of heavy metals in other organelles. The present study indicates that CHI I may play an important role in the processes of heavy metal homeostasis and possibly detoxification. In Figure 7, we will learn the mechanism of chitinase resistance to heavy metals. The expression characteristics of different mangrove plant chitinase genes under complex heavy metal stress were characterized, and the analysis results showed that there was variability in the expression pattern characteristics, while the expression of mangrove plant chitinase genes changed more significantly under the conditions of complex heavy metal stress, showing that this gene plays an important role in mangrove plants’ ability to resist heavy metal stress. It also showed that different types of chitinase genes play different roles in different mangrove plants and allowed the prediction that different subtypes of chitinase have different sites of action in cells [60]. Different types of chitinases can be isolated in mangrove species, and there are large differences in structure [60]. The structure determines the functional role, with type I chitinase playing a functional role inside the vacuole, while type Ⅲ chitinase acts outside the cell. Two expression patterns emerged from the combined analysis: the expression changes trended upward and then downward or upward from the analysis on the concentration of heavy metal stress; the expression changes trended upward and then downward or upward as the time of heavy metal stress was prolonged. The expression of mangrove plants showed different responses to heavy metal stress, and the expression trend rose and then declined, indicating that the plants themselves have a certain tolerance, and the plants to withstand less with too high a heavy metal concentration or too long a stress time. The specific expression of the type I chitinase gene showed the gene expression of chitinase in *B. gymnorrhiza* > *A. marina* > *K. obovata* > *R. stylosa*. The gene expression of chitinase was more highly induced in *B. gymnorrhiza* leaves than in other mangrove plant species. Mangroves, as more tolerable species to heavy metal, can be used as a potential phytoremediator in heavy-metal-polluted marine wetlands.

## 4. Materials and Methods

### 4.1. Plant Material and Treatments

The six-month-old seedlings of *Rhizophora stylosa*, *Bruguiera gymnorrhiza*, *Kandelia obovata*, and *Avicennia marina* were collected from Zhanjiang City, Guangdong Province, China. We planted 3 seedlings of each of the above species in each pot and divided them into 5 pots filled with sand (control group, CK; C1; C2; C3; and C4). Each pot was irrigated with 500 mL of 1/2 Hoagland solution (containing 10% NaCl) every 3 days. The plants were watered with heavy metal sewage (pH = 6), which was artificially prepared in five different concentrations (Table 2). Fresh leaves of plants were collected after 0 days, 3 days, 7 days, 14 days, and 28 days under heavy metal treatment (samples were used in three replicates). All the collected samples were immediately frozen in liquid nitrogen and stored at −80 °C before use.

### 4.2. Total RNA Isolation and First-Strand cDNA Synthesis

Total RNA was extracted from leaves via the centrifuging column method using the Tiangen polysaccharide polyphenol plant total RNA extraction kit, following the manufacturer’s protocol. Total RNA was dissolved in 30 µL of RNase-free water. Total RNA was quantified via spectrometry, and quality was checked on denatured agarose gels. First-strand cDNA was synthesized using PrimeScript TM Reverse Transcriptase (Takara, Dalian, China) following the manufacturer’s instructions. Total RNA with 10 mM dNTP in a total volume of 20 µL by incubating for 5 min at 65 °C, 1 h at 50 °C, and 5 min at 85 °C in accordance with the manufacturer’s instructions. First-strand cDNA was stored at −20 °C before use.

### 4.3. Cloning the Full-Length cDNA of Chitinase Gene

The sequences of primers are shown in Table 3. According to the conserved sequence of the chitinase gene in other homologous species, the primers (F1 and R1) of the intermediate fragment were designed, and the intermediate fragment was amplified. To obtain a full-length cDNA, two gene-specific primers (GSP1, GSP2) and two nested PCR primers (NGSP1 and NGSP2) were deduced from the internal cDNA fragment. Then, 5′-RACE and 3′-RACE PCR procedures were performed using SMARTer TM RACE Kit (Clontech, WI, USA) in accordance with the manufacturer’s instructions. qF and qR are the primers used for the experimental cloning of full-length genes.

### 4.4. Bioinformatic Analysis

The full-length cDNA sequence was analyzed using ApE software and ORF-Finder. Molecular weight, theoretical pI, and amino acid composition were analyzed using the Prot-Param tool. Homology searches were carried out using the NCBI BLAST server. Subcellular localization of proteins using Plant-mpLoc analysis. The SWISS-MODEL (http://swissmodel.expasy.org (accessed on 6 May 2021).) was used to generate homology modeling of the structure of CHI. The phylogenetic tree was constructed using the MEGA 6.0 package using the neighbor-joining algorithm with bootstrap analyses for 1000 replicates.

### 4.5. Analysis of CHI I Gene Expression by Real-Time Quantitative PCR

Real-time RT-PCR reactions were performed on heavy metal from three replicates in leaf tissues per treatment, performed in twofold replicates for each sample. The amplicon size is 153 nt, the primer Tm is 29 °C and the primer efficiency is 91% for real-time PCR primers *R. stylosa* 18S rRNA (GenBank accession No. AY289627.1), *B. gymnorrhiza* 18S rRNA (GenBank accession No. AB233615.1), *K. obovata* 18S rRNA (GenBank accession No. AY289625.1), and *A. marina* 18S rRNA (GenBank accession No. AY289627.1) were used as housekeeping reference genes to normalize the expression levels between samples. All data were given in terms of relative mRNA expressed as the mean ± SD. The Dunnett’s multiple comparison test (*p* < 0.05) was used to evaluate differences between means of treatment using SPSS 22.0 software.

## 5. Conclusions

Four new type I chitinase genes (*CHI*) were cloned from *Bruguiera gymnorrhiza*, *Rhizophora stylosa*, *Kandelia obovata*, and *Avicennia marina*. The type I chitinase structure includes a signal peptide region at its N-terminus, a chitin-binding domain (CBD), and a glycosyl hydrolase catalytic domain, and *CHI I* belongs to glycosidase family 19. Although the four cDNAs had a full length of 1092 bp and an ORF (open reading frame) of 831 bp, coding for 276 amino acids, they had different gene sequences among them. Furthermore, it was also indicated that the *CHI I* transcripts were differentially expressed in four mangrove species under heavy metal. The gene expression of *CHI I* was more highly induced in *B. gymnorrhiza* leaves than in other mangrove species. The *CHI I* protein is an unstable hydrophilic protein, mainly distributed in intracellular vacuoles. This study will provide more details on the molecular mechanisms or a scientific basis for coastal wetland heavy metal environmental remediation with mangrove plants.

## Figures and Tables

**Table 1:** Physical and chemical properties of Chi I.

Species	Name of Gence	Number of Amino Acids	Molecular Weight	pI	The High Content of Amino Acid	Instability Index	Stable or Not	Grand Average of Hydropathicity
*Rhizophora stylosa*	*Rs Chi*	276aa	29.47 kDa	4.65	Gly11.2% Ala9.1% Ser 9.1%	27.79	Y	−0.186
*Bruguiera gymnorrhiza*,	*Bg Chi*	276aa	29.50 kDa	4.74	Gly11.2% Ser 9.1% Ala9.1%	27.79	Y	−0.189
*Kandelia obovata*,	*Ko Chi*	276aa	29.59 kDa	4.69	Gly11.2% Ser 9.1% Ala8.7%	27.16	Y	−0.218
*Avicennia marina*	*Am Chi*	276aa	25.57 kDa	4.66	Gly10.9% Ser 9.1% Ala8.3%	28.75	Y	−0.155

**Table 2:** Heavy metal concentrations in artificial sewage prepared from 1/2 Hoagland nutrient solution.

Heavy Metal (mg/L)	Control Group (CK)	C1	C2	C3	C4
Cu^2+^	0	5.0	25.0	50.0	75.0
Pb^2+^	0	1.0	5.0	10.0	15.0
Cd^2+^	0	0.2	1.0	2.0	3.0

**Table 3:** List of primers for PCR, RACE, and real-time PCR experiments.

Primers	Sequence (5′–3′)
F1	GGCTCCTTCACTTATTCACG
R1	ATTGTCTCCCCAAACCCT
GSP1	ATTGTCTCCCCAAACCCT
GSP2	GCTCCTTCACTTATTCACG
NGSP1	GCAAGAGTGAGAGATAGCGAAGGTT
NGSP2	GATACAACTGTCCTGGAACTT
qF	GTGGCACAGGCAGTGAATAC
qR	CCTTCCCCTCGCAACTAG
Bg18S (F)	CGGGGGCATTCGTATTTC
Bg18S (R)	CCTGGTCGGCATCGTTTAT
Ko18S (F)	CCTGAGAAACGGCTACCACATC
Ko18S (R)	ACCCATCCCAAGGTCCAACTAC
Am18S (F)	CCCGTTGCTGCGATGAT
Am18S (R)	GCTGCCTTCCTTGGATGTG
Rs18S (F)	ACCATAAACGATGCCGACC
Rs18S (R)	CCTTGCGACCATACTCCC

## Data Availability

The data presented in this study are available on request from the corresponding author.

## Supplementary Materials

The following supporting information can be downloaded at https://www.mdpi.com/article/10.3390/plants12152772/s1. Figure S1: The nucleotide sequence of the full-length cDNA and the deduced amino acid sequence of the CHI I gene. The start codon ATG and stop codon TAG are shown in bold italics, and the predicted amino acid sequence is shown in a one-letter code under the DNA sequence. The AATAA box is highlighted in gray, and the poly (A) tail is highlighted in gray (a: BgChi, b: KoChi, c: AmChi, d: RsChi). Figure S2: Comparison of chitinase gene sequences among four mangrove species (red markers show differences in four full-length genes).
