# Supplementary material for: Cloning and Expression of Class I Chitinase Genes from Four Mangrove Species under Heavy Metal Stress
Source: Plants (Basel). 2023 Jul 26;12(15):2772. doi: 10.3390/plants12152772 (PMC10421288; doi:10.3390/plants12152772)

The supplement data.

Figure S1: The nucleotide sequence of the full-length cDNA and the deduced amino acid sequence of the CHI I gene. The start codon ATG and stop codon TAG are shown in bold italic, the predicted amino acid sequence is shown in one-letter code under the DNA sequence. The AATAA-box is highlighted in gray, the poly (A) tail is highlighted in gray (a: *BgChi*, b: *KoChi*, c: *AmChi*, d: *RsChi*)

>a

1 ***ATG***GCTCCTTCACTTATTCACGTCAAACCTTCGCTATCTCTCACTCTTGCGGCAATCTTT

1 M  A  P  S  L  I  H  V  K  P  S  L  S  L  T  L  A  A  I  F

61 CTCTCCGCCGTAGCTCCAGTTTTCAGCCAAAACTGTGGCTGCGCACCGGACTTGTGCTGC

21 L  S  A  V  A  P  V  F  S  Q  N  C  G  C  A  P  D  L  C  C

121 AGCCAGTGGGGTTACTGTGGCACAGGCAGTGAATACTGCGGCCAAGGCTGCCAGTCAGGG

41 S  Q  W  G  Y  C  G  T  G  S  E  Y  C  G  Q  G  C  Q  S  G

181 CCATGCTCCGGCCCGCCCAGCGGCAATGGCGTTTCCGTTGCCGACATCGTGACCGACGCG

61 P  C  S  G  P  P  S  G  N  G  V  S  V  A  D  I  V  T  D  A

241 TTCTTTAACGGGATCGCGGATCAAGCTGCGGCTAGTTGCGAGGGGAAGGGATTCTACACT

81 F  F  N  G  I  A  D  Q  A  A  A  S  C  E  G  K  G  F  Y  T

301 CGCGCTGCCTTTCTGGAGGCGCACGGTTCGTATTCCCAGTTCGGTACGGTGGGTTCGTCT

101 R  A  A  F  L  E  A  H  G  S  Y  S  Q  F  G  T  V  G  S  S

361 GATGATTCGAAGCGTGAGATCGCAGCCTTTTTTGCTCACGTCACCCATGAGACTGGACAT

121 D  D  S  K  R  E  I  A  A  F  F  A  H  V  T  H  E  T  G  H

421 ATGTGCTACATTGAAGAGATCAACGGCTCGTCTGGAGACTACTGCGACGAAACCAACACG

141 M  C  Y  I  E  E  I  N  G  S  S  G  D  Y  C  D  E  T  N  T

481 CAGTATCCATGCGCTCCCAACAAGGAATATTATGGTCGAGGGCCGATACAACTGTCCTGG

161 Q  Y  P  C  A  P  N  K  E  Y  Y  G  R  G  P  I  Q  L  S  W

541 AACTTCAACTATGGGCCTGCAGGCAATAGCATAGGTTTCGATGGCTTAAAGAATCCAGAA

181 N  F  N  Y  G  P  A  G  N  S  I  G  F  D  G  L  K  N  P  E

601 ATTGTGGCCACAGATCCAGTCATATCCTTCAAGACTGCACTGTGGTATTGGATGAATAAT

201 I  V  A  T  D  P  V  I  S  F  K  T  A  L  W  Y  W  M  N  N

661 TGCCATGATCTAATAATTTCGGGACAAGGGTTTGGGGCAACAATTCGTGCCATTAATGGT

221 C  H  D  L  I  I  S  G  Q  G  F  G  A  T  I  R  A  I  N  G

721 CGACTTGAATGTGATGGGGCCAACCCCAACACTGTTAGTTCTCGTGTCGAGTATTATACA

241 R  L  E  C  D  G  A  N  P  N  T  V  S  S  R  V  E  Y  Y  T

781 CAATATTGCAACCAGCTCCAAGTTGATCCTGGTAATAATTTAAGATGC***TAG***TATAACGGG

261 Q  Y  C  N  Q  L  Q  V  D  P  G  N  N  L  R  C  *

841 GATCGTGCTTGAAAGGCGATGTCATAGCTTATGGGAATTTTATATCTAATGAAATGAATG

901 ATTATAAGGATCATGTAAGCTGTCTGTAGTCTTGTTCGAACGTATGATTATAGGTTTTGG

961 TGGTGTAATAATTTATAAAGCACGTCTATAGTGCAAAATAATGTATAAAGTATGTCTCCC

1021 AATTGGTAATTGTACCCGAAATATAATAATTTGCCTAAATATTGACCTTTTTATAAAAAA

1081 AAAAAAAAAAAA

>b

1 ***ATG***GCTCCTTCACTTATTCACGTCAAACCTTCGCTATCTCTCACTCTTGCGGCAATCTTT

1 M  A  P  S  L  I  H  V  K  P  S  L  S  L  T  L  A  A  I  F

61 CTCTCCGCCGTAGCTCCAGTTTTCAGCCAAAACTGTGGCTGCGCACCGGACTTGTGCTGC

21 L  S  A  V  A  P  V  F  S  Q  N  C  G  C  A  P  D  L  C  C

121 AGCCAGTGGGGTTACTGTGGCACAGGCAGTGAATACTGCGGCCAAGGCTGCCAGTCAGGG

41 S  Q  W  G  Y  C  G  T  G  S  E  Y  C  G  Q  G  C  Q  S  G

181 CCATGCTCCGGCCCGCCCAGCGGCAATGGCGTTTCCGTTGCCGACATCGTGACCGACGCG

61 P  C  S  G  P  P  S  G  N  G  V  S  V  A  D  I  V  T  D  A

241 TTCTTTAACGGGATCGCGGATCAAGCTGCGGCTAGTTGCGAGGGGAAGGGATTCTACACT

81 F  F  N  G  I  A  D  Q  A  A  A  S  C  E  G  K  G  F  Y  T

301 CGCGCTGCCTTTCTGGAGGCGCACGGTTCGTATTCCCAGTTCGGTACGGTGGGTTCGTCT

101 R  A  A  F  L  E  A  H  G  S  Y  S  Q  F  G  T  V  G  S  S

361 GATGATTCGAAGCGTGAGATCGCAGCCTTTTTTGCTCACGTCACCCATGAGACTGGACAT

121 D  D  S  K  R  E  I  A  A  F  F  A  H  V  T  H  E  T  G  H

421 ATGTGCTACATTGAAGAGATCAACGGCTCGTCTGGAGACTACTGCGACGAAACCAACACG

141 M  C  Y  I  E  E  I  N  G  S  S  G  D  Y  C  D  E  T  N  T

481 CAGTATCCATGCGCTCCCAACAAGGAATATTATGGTCGAGGGCCGATACAACTGTCCTGG

161 Q  Y  P  C  A  P  N  K  E  Y  Y  G  R  G  P  I  Q  L  S  W

541 AACTTCAACTATGGACCTGCAGGCAATAGCATAGGTTTCGATGGCTTAAACAATCCAGAA

181 N  F  N  Y  G  P  A  G  N  S  I  G  F  D  G  L  N  N  P  E

601 ATTGTGGCCACAGATCGAGTCGTATCTTTCAAGACTGCACTGTGGTATTGGATGAATAAT

201 I  V  A  T  D  R  V  V  S  F  K  T  A  L  W  Y  W  M  N  N

661 TGCCATGATCTAATAATTTCGGGACAAGGGTTTGGGGAGACAATTCGTGCCATTAATGGT

221 C  H  D  L  I  I  S  G  Q  G  F  G  E  T  I  R  A  I  N  G

721 CGACTTGAATGTGATGGGGCCAACCCCAACACAGTTAGTTCTCGTGTCGAGTATTATACA

241 R  L  E  C  D  G  A  N  P  N  T  V  S  S  R  V  E  Y  Y  T

781 CAATATTGCAACCAGCTCCAAGTTGATCCTGGTAATAATTTAAGATGC***TAG***TATAACGGG

261 Q  Y  C  N  Q  L  Q  V  D  P  G  N  N  L  R  C  *

841 GATCGTGCTTGAAAGGCGATGTCATAGCTTATGGGAATTTTATATCTAATGAAATGAATG

901 ATTATAAGGATCATGTAAGCTGTCTGTAGTCTTGTTCGAACGTATGATTATAGGTTTTGG

961 TGGTGTAATAATTTATAAAGCACGTCTATAGTGCAAAATAATGTATAAAGTATGTCTCCC

1021 AATTGGTAATTGTACCCGAAATATAATAATTTGCCTAAATATTGACCTTTTTATAAAAAA

1081 AAAAAAAAAAAA

>c

       1 ***ATG***GCTCCTTCACTTATTCACGTCAAACCTTCGCTATCTCTCACTCTTGCGGCAATCTTT

       1 M  A  P  S  L  I  H  V  K  P  S  L  S  L  T  L  A  A  I  F

      61 CTCGTCGCCGTAGCTCCAGTTTTCAGCCAAAACTGTGGCTGCGCACCGGACTTGTGCTGC

      21 L  V  A  V  A  P  V  F  S  Q  N  C  G  C  A  P  D  L  C  C

     121 AGCCAGTGGGGTTACTGCGGCAAAGGCAGTGAATACTGCGGCCAAGGCTGCCAGTCGGGG

      41 S  Q  W  G  Y  C  G  K  G  S  E  Y  C  G  Q  G  C  Q  S  G

     181 CCATGCTCCGGCACGCCCAGCGGCAATGGCGTTTCCGTTGCTGATATCGTGACCGACGCG

      61 P  C  S  G  T  P  S  G  N  G  V  S  V  A  D  I  V  T  D  A

     241 TTCTTTAACGGGATCGCGGATCAAGCTGCGGCAAGTTGCGAGGGGAAGGGATTCTACACT

     81 F  F  N  G  I  A  D  Q  A  A  A  S  C  E  G  K  G  F  Y  T

     301 CGCGCTGTCTTTCTGGAGGCGCTCGGTTCGTATTCCCAGTTCGGTACGGTGGGTTCGTCT

     101 R  A  V  F  L  E  A  L  G  S  Y  S  Q  F  G  T  V  G  S  S

     361 GATGATTCGAAGCGTGAGATCGCCGCCTTTTTCGCTCACGTCACCCATGAGACTGGACAT

     121 D  D  S  K  R  E  I  A  A  F  F  A  H  V  T  H  E  T  G  H

     421 ATGTGCTCCATTGAAGAGATCAACGGCTCATCAAGAGACTACTGCGACGAGAACAACACA

     141 M  C  S  I  E  E  I  N  G  S  S  R  D  Y  C  D  E  N  N  T

     481 CAGTATCCCTGCGTTCCCAACAAGGAATATTATGGTCGAGGGCCGATACAACTGTCCTGG

     161 Q  Y  P  C  V  P  N  K  E  Y  Y  G  R  G  P  I  Q  L  S  W

     541 AACTTCAACTATGGGCCTGCAGGCAATAGCATAGGTTTCGATGGCTTAAACAATCCAGAA

     181 N  F  N  Y  G  P  A  G  N  S  I  G  F  D  G  L  N  N  P  E

     601 ATTGTGGCCACAGATCCAGTCATATCTTTCAAGACTGCACTGTGGTATTGGATGAATAAT

     201 I  V  A  T  D  P  V  I  S  F  K  T  A  L  W  Y  W  M  N  N

     661 TGCCATGATCTAATAATTTCGGGACAAGGGTTTGGGGCTACAATTCGTGCCATTAATGGT

     221 C  H  D  L  I  I  S  G  Q  G  F  G  A  T  I  R  A  I  N  G

     721 CAACTTGAATGTGATGGGGCCAACCCCAACACTGTTAGTTCTCGCGTCGAGTATTATACA

     241 Q  L  E  C  D  G  A  N  P  N  T  V  S  S  R  V  E  Y  Y  T

     781 CAATATTGCAACCAGCTTCAAGTTGATCCTGGTAATAATTTGAGATGC***TAG***TATAACGTG

     261 Q  Y  C  N  Q  L  Q  V  D  P  G  N  N  L  R  C  *

841 GATCGTGCTTGAAAGGCGATGTCATAGCTTATGGGAATTTTATATCTAGTGAAATGAATG

     901 ATTATAAGGATCATGTAAGCTGTCTGTAGTCTTGTTCGAACGTATGATTATAGGTTTTGG

     961 TGGTGTAATAATTTATAAAGCACGTCTATAGTGCAAAATAATGTATAAAGTATGTCTCCC

    1021 AATTGGTAATTGTACCCGAAATATAATAATTTGCCTAAATATTGACCTTTTTATAAAAAA

    1081 AAAAAAAAAAAA

>d

       1***ATG***GCTCCTTCACTTATTCACGTCAAACCTTCGCTATCTCTCACTCTTGCGGCAATCTTT

      1 M  A  P  S  L  I  H  V  K  P  S  L  S  L  T  L  A  A  I  F

      61 CTCTCCGCCGTAGCTCCAGTTTTCAGCCAAAACTGTGGCTGCGCACCGGACTTGTGCTGC

      21 L  S  A  V  A  P  V  F  S  Q  N  C  G  C  A  P  D  L  C  C

     121 AGCCAGTGGGGTTACTGTGGCACAGGCAGTGAATACTGCGGCCAAGGCTGCCAGTCAGGG

      41 S  Q  W  G  Y  C  G  T  G  S  E  Y  C  G  Q  G  C  Q  S  G

     181 CCATGCTCCGGCCCGCCCAGCGGCAATGGCGTTTCCGTTGCCGACATCGTGACCGACGCG

      61 P  C  S  G  P  P  S  G  N  G  V  S  V  A  D  I  V  T  D  A

     241 TTCTTTAACGGGATCGCGGATCAAGCTGCGGCTAGTTGCGAGGGGAAGGGATTCTACACT

      81 F  F  N  G  I  A  D  Q  A  A  A  S  C  E  G  K  G  F  Y  T

     301 CGCGCTGCCTTTCTGGAGGCGCACGGTTCGTATTCCCAGTTCGGTACGGTGGGTTCGTCT

     101 R  A  A  F  L  E  A  H  G  S  Y  S  Q  F  G  T  V  G  S  S

     361 GATGATTCGAAGCGTGAGATCGCAGCCTTTTTTGCTCACGTCACCCATGAGACTGGACAT

     121 D  D  S  K  R  E  I  A  A  F  F  A  H  V  T  H  E  T  G  H

     421 ATGTGCTACATTGAAGAGATCAACGGCTCGTCTGGAGACTACTGCGACGAAACCAACACG

     141 M  C  Y  I  E  E  I  N  G  S  S  G  D  Y  C  D  E  T  N  T

     481 CAGTATCCATGCGCTCCCAACAAGGAATATTATGGTCGAGGGCCGATACAACTGTCCTGG

     161 Q  Y  P  C  A  P  N  K  E  Y  Y  G  R  G  P  I  Q  L  S  W

     541 AACTTCAACTATGGACCTGCAGGCAATAGCATAGGTTTCGATGGCTTAAAAAATCCAGAA

     181 N  F  N  Y  G  P  A  G  N  S  I  G  F  D  G  L  K  N  P  E

     601 ATTGTGGCCACAGATCCAGTCATATCCTTCAAGACTGCACTGTGGTATTGGATGAATAAT

     201 I  V  A  T  D  P  V  I  S  F  K  T  A  L  W  Y  W  M  N  N

     661 TGCCATGATCTAATAATTTCGGGACAAGGGTTTGGGGCTACAATTCGTGCCATTAATGGT

     221 C  H  D  L  I  I  S  G  Q  G  F  G  A  T  I  R  A  I  N  G

     721 CAACTTGAATGTGATGGGGCCAACCCCAACACAGTTAGTTCTCGCGTCGAGTATTATACA

     241 Q  L  E  C  D  G  A  N  P  N  T  V  S  S  R  V  E  Y  Y  T

     781 CAATATTGCAACCAGCTCCAAGTTGATCCTGGTAATAATTTAAGATGC***TAG***TATAACGGG

     261 Q  Y  C  N  Q  L  Q  V  D  P  G  N  N  L  R  C  *

841 GATCGTGCTTGAAAGGCGATGTCATAGCTTATGGGAATTTTATATCTAATGAAATGAATG

     901 ATTATAAGGATCATGTAAGCTGTCTGTAGTCTTGTTCGAACGTATGATTATAGGTTTTGG

     961 TGGTGTAATAATTTATAAAGCACGTCTATAGTGCAAAATAATGTATAAAGTATGTCTCCC

    1021 AATTGGTAATTGTACCCGAAATATAATAATTTGCCTAAATATTGACCTTTTTATAAAAAA

    1081 AAAAAAAAAAAA

Figure S2: Comparison of chitinase gene sequences among four mangrove species (red markers show differences in four full-length genes).


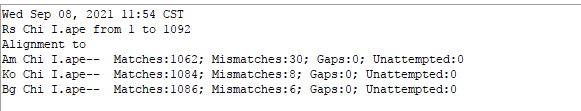

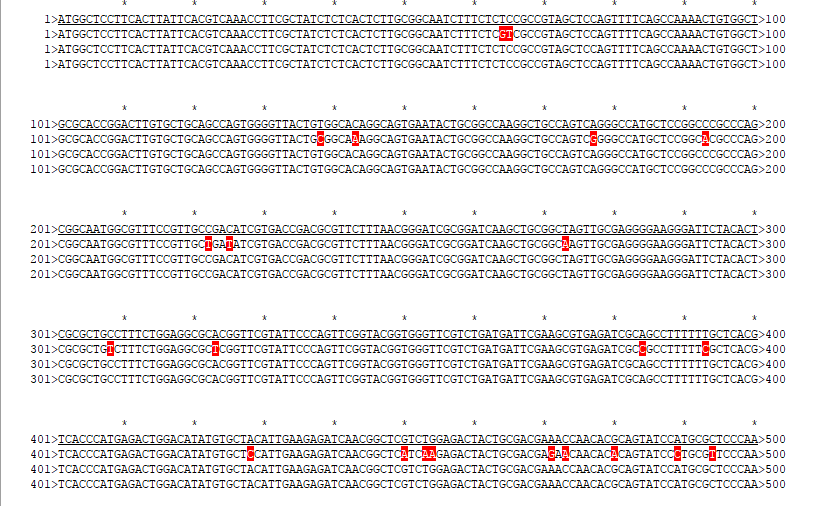

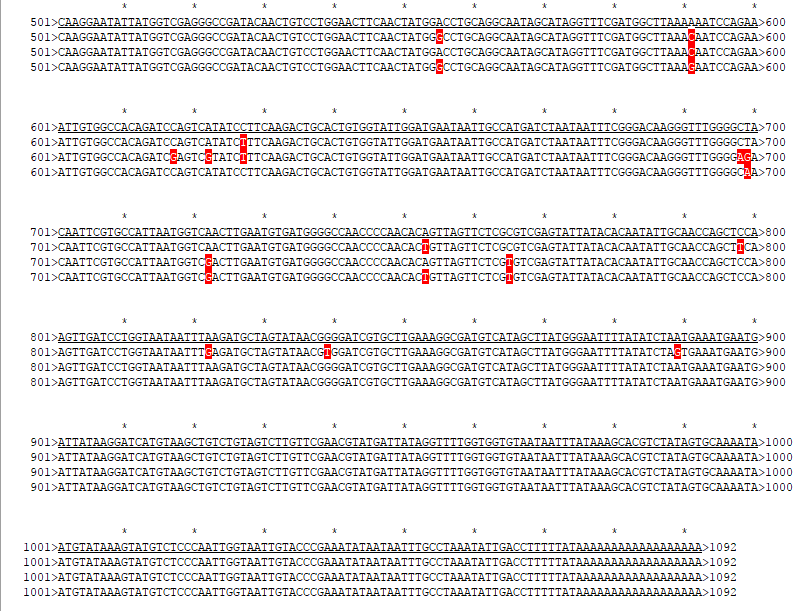

Supplement: Supplementary file 1 [file plants-12-02772-s001.zip › plants-2422404-supplementary.docx]
